# Efficacy of hydrofibre dressing following total joint arthroplasty: a meta-analysis of randomised controlled trials

**DOI:** 10.1177/11207000211012669

**Published:** 2021-05-02

**Authors:** Raman Mundi, Harman Chaudhry, Seper Ekhtiari, Prabjit Ajrawat, Daniel M Tushinski, Thomas J Wood, Mohit Bhandari

**Affiliations:** 1 Division of Orthopaedic Surgery, Department of Surgery, University of Toronto, Toronto, ON, Canada; 2 Division of Orthopaedic Surgery, Department of Surgery, McMaster University, Hamilton, ON, Canada

**Keywords:** infection, arthroplasty, hydrofiber dressing

## Abstract

**Introduction:**

In the United States, over 1,000,000 total joint arthroplasty (TJA) surgeries are performed annually and has been forecasted that this number will exceed 4,000,000 by the year 2030. Many different types of dressing exist for use in TJA surgery, and it is unclear if any of the newer, hydrofibre dressings are superior to traditional dressings at reducing rates of infections or improving wound healing. Thus, the aim of this systematic review and meta-analysis was to assess the impact of hydrofiber dressings on reducing complications.

**Methods:**

A systematic review and meta-analysis was performed using the online databases MEDLINE and the Cochrane Library. Randomized controlled trials (RCTs) comparing hydrofibre dressings to a standard dressing were included. Summary measures are reported as odds ratios (ORs) and mean differences (MDs) with 95% confidence intervals (CIs). Our primary outcome was prosthetic joint infection (PJI). Secondary outcomes included blisters, dressing changes and wound irritation.

**Results:**

5 RCTs were included. Hydrofibre dressing had no observable effect on PJI or wound irritation (OR 0.53; 95% CI, 0.14–1.98; *p* = 0.35). Hydrofibre dressings reduced the rate of blisters (OR 0.36; 95% CI, 0.14–0.90; *p* = 0.03) and number of dressing changes (MD -1.89; 95% CI, -2.68 to -1.11).

**Conclusions:**

In conclusion, evidence suggests hydrofibre dressings have no observable effect on PJI and wound irritation. Evidence for reduction in blisters and number of dressings is modest given wide CIs and biased trial methodologies. Use of hydrofibre dressings should be considered inconclusive for mitigating major complications in light of current best evidence.

## Introduction

Annually, over 120,000 primary hip and knee arthroplasty procedures are performed in Canada.^1^ In the United States, >1,000,000 are performed annually and has been forecasted that this number will exceed 4,000,000 by the year 2030.^2–4^ Despite the overall positive outcomes with joint replacement surgery, wound complications and periprosthetic joint infections (PJIs) remain 1 of the most dreaded complications of this procedure.^5,6^ It is estimated that the overall risk of PJI is 1.4%.^7^ Given the morbidity and mortality associated with infections after total joint arthroplasty (TJA), any modifiable treatment factors that can possibly mitigate such complications are invariably sought by surgeons and hospitals with considerable interest. Furthermore, given the magnitude of joint replacement procedures performed nationally and globally, even incremental improvements in wound complications and infection risk can have substantial impact on patient care and resources.

Several types of wound dressings are available for routine use in hip and knee arthroplasty procedures, which all vary in cost, comfort, and purported efficacy in mitigating wound complications. A relatively new type of dressing, known as hydrofibre dressings, have become increasingly popular given its reported ability to reduce surgical site infections, skin blistering, and frequency of dressing changes.^8^ These dressings achieve such outcomes purportedly through their hydrofibre technology. Some dressings are also silver impregnated, which theoretically contributes to antibacterial properties. The hydrofibre material absorbs wound exudates and transforms it into a gel that creates an optimal environment for wound healing. The dressing also has an outer hydrocolloid perimeter which is impermeable. Small randomised trials have suggested advantages to this type of dressing; however, their lack of study power and resultant wide confidence intervals have rendered its efficacy uncertain. Thus, the aim of this systematic review and meta-analysis was to review the available literature on hydrofibre dressings and specifically, to assess its impact on rates of deep PJIs, dressing changes, wound irritation, and blister formation.

## Methods

This systematic review and meta-analysis was conducted in accordance with the Cochrane Handbook for Systematic Reviews of Interventions Version 6.0 and reported according to the Preferred Reporting Items for Systematic Reviews and Meta-Analyses (PRISMA) guidelines.^9,10^

### Eligibility criteria

Studies meeting the following criteria were included: (1) randomised controlled trials; (2) adult patients (⩾18 years); (3) undergoing primary total hip (THA) or knee arthroplasty (TKA); (4) assessing a hydrofibre dressing compared to a standard dressing; (5) reporting 1 or more of the following outcomes: wound complications, PJIs, frequency of dressing changes; (6) full text available in English through the McMaster University library; and (7) published in the last 10 years. Studies exclusively including revision THA or TKA were excluded.

### Information sources and search

Through the OVID interface, the following bibliographic databases was systematically searched: Medline Epub Ahead of Print, In-Process & Other Non-Indexed Citations, MEDLINE(R) Daily, MEDLINE(R) (1946 – Present). The Cochrane Library database was also searched (Issue 11, 2019). The search strategy in both databases consisted of keywords and controlled vocabulary, in the form of the National Library of Medicine’s MeSH terms (Medical Subject Headings). The primary search themes were arthroplasty hip, arthroplasty knee, aquacel and hydrofibre. Grey literature was identified through various sources, including the website of HTA agencies (CADTH), clinical trials registries (ClinicalTrials.gov), reference lists of published reviews, proceedings from national association meetings (Canadian Orthopaedic Association), and online search engines (Google Scholar). The search was executed on 01 December 2019.

### Study selection

Screening was performed in duplicate at the title/abstract and full text stages. At the title/abstract stage, any disagreements were resolved by automatic inclusion in the next round. At the full text stage, any disagreements were resolved by discussion until consensus.

### Data collection process and data items

Data was extracted in duplicate by 2 reviewers (RM and PA) and entered into a Microsoft Excel spreadsheet (Microsoft, Redmond, Washington, USA). Any discrepancies were reviewed and resolved via consensus. Data variables to be collected were determined *a priori*, and included: (1) study characteristics (year of publication, country, number of centres, sample size, procedure [THA, TKA, Both]); (2) patient characteristics (mean age, number of males/females); (3) treatment characteristics (intervention dressing, control dressing, surgical approach, wound closure method, antibiotic prophylaxis, drain usage); and (4) outcomes (follow-up duration, number of patients with blisters, wound irritation, PJIs, and number of dressing changes).

### Risk of bias in individual studies

Quality assessment was conducted using the Cochrane Risk of Bias Assessment Tool applied to each individual trial.^11^

### Summary measures and synthesis of results

Baseline study, demographic, and treatment characteristics are presented using descriptive statistics. Categorical data is presented as frequencies with associated percentages. Continuous data is presented as means with associated standard deviations or medians with associated interquartile ranges, depending on what was reported in the original trial publication.

For the outcomes of interest including blisters, wound irritation, infections, and dressing changes, we calculated pooled effect estimates across trials using Review Manager Software (RevMan 5.3). In the event of missing data (i.e. standard deviations), this was obtained through previously published reports or derived by computational methods. Pooled effect estimates are present as odds ratios (ORs) for categorical outcomes and mean differences (MDs) for continuous variable with associated 95% confidence intervals (CIs). A *p*-value < 0.05 was established *a priori* as statistically significant. Heterogeneity was assessed using the chi-square test (*p* < 0.1 established as significance threshold) and the I^2^ statistic. Random effects models were used for all analyses irrespective of heterogeneity.

## Results

### Study selection

**Figure 1.**
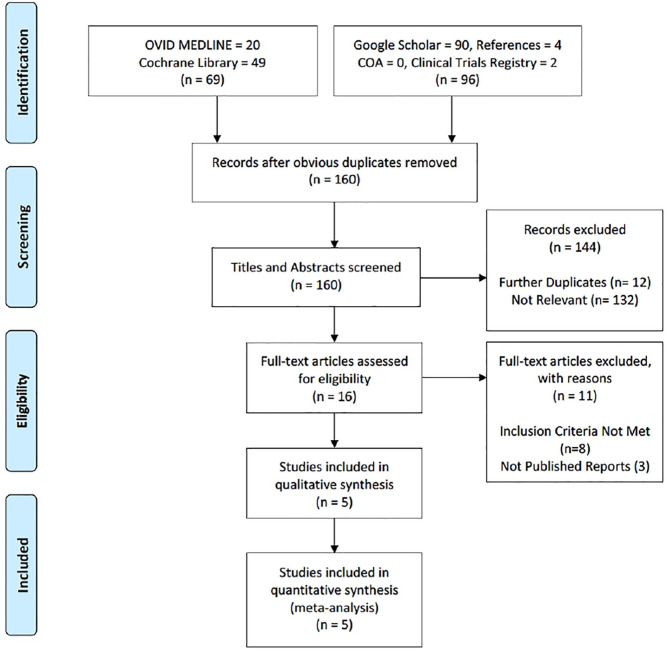
PRISMA flow diagram for clinical review.

The abovementioned search strategy yielded 165 citations, including 69 through the two database searches and 96 through the grey literature search. After removal of obvious duplicates, a total of 160 titles and abstracts were screened of which 16 were included for full-text review. 5 articles met the inclusion criteria and were included for both qualitative and quantitative synthesis.^12–16^ Results of the study screening and selection are outline in Figure 1.

### Study characteristics

The 5 included studies were published between 2011 and 2017. They were all single-centre trials and had sample sizes ranging from 80 to 262. 2 of the studies exclusively evaluated patients undergoing TKA, whereas 3 of the studies evaluated both TKA and THA procedures.

The mean age of patients in each trial ranged from 63 to 72 years, and only 1 trial had more male patients than female patients. All studies evaluated the same hydrofibre dressing (Aquacel) as the intervention but varied in terms of the specific comparator dressing. Furthermore, 2 of the 5 trials used silver-impregnated Aquacel Ag,^14,16^ whereas the remaining 3 trials used Aquacel with no silver.^12,13,15^ 4 of the 5 trials had follow-up of 30 days or less, whereas 1 trial had follow-up to 1 year. Details of the trials are presented in Appendix Tables 1 and 2.

### Risk of bias within studies

**Figure 2.**
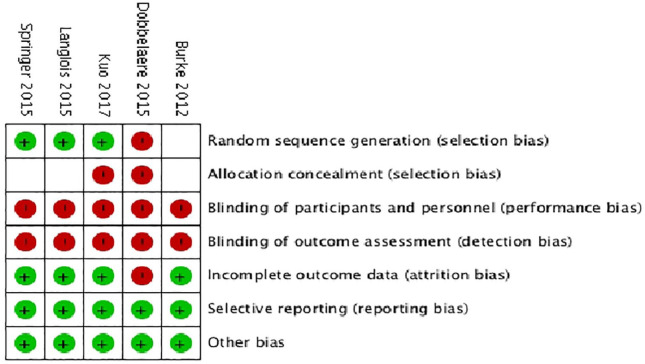
Cochrane risk of bias tool.

Overall, the methodological quality of the studies was poor and they were deemed high risk of bias (Figure 2). 3 studies reported an appropriate method of randomisation sequence generation. No studies were low risk of bias in terms of allocation concealment, blinding of participants or blinding or outcome assessors. The latter 2 were not feasible given the nature of the intervention. 4 of the 5 studies were low risk of bias for incomplete outcome data.

### Synthesis of results

#### Periprosthetic joint infection

Only 2 studies defined their criteria for infection: 1 study defined it as an “erythematous, indurated wound with copious persistent discharge”,^12^ whereas the other study used the Centers for Disease Control (CDC) definition.^14^ 4 of the 5 studies reported having no PJIs across both treatment groups. 1 trial that had follow-up of 1 year, found a single infection in the control group.^14^ A pooled analysis was not performed for this outcome as there was only 1 event in either group.

#### Blisters

**Figure 3.**
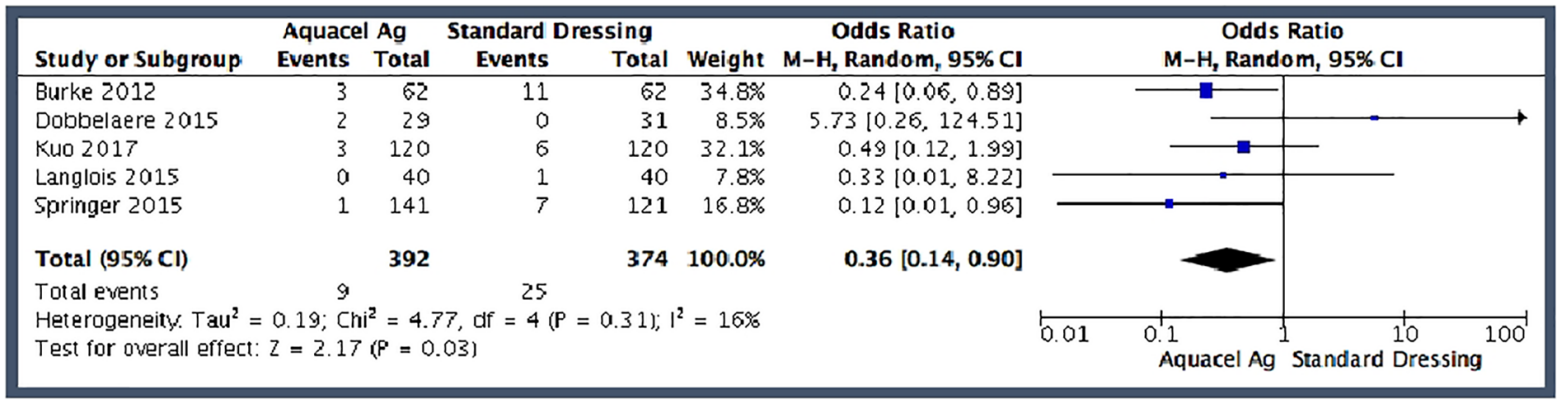
Pooled analysis for blisters.

All 5 studies reported the risk of wound blisters, and as such, were all included in the pooled analysis. The pooled rate of blisters was 2.3% in the hydrofibre dressing group (*n* = 9 of 392) and 6.7% in the standard dressing group (*n* = 25 of 374). The pooled effect estimate demonstrated a statistically significant decrease in the odds of blisters with the use of the hydrofibre dressing (OR 0.36; 95% CI, 0.14–0.90) (Figure 3). Heterogeneity across the studies was low (I^2^ = 16%) and not significant (chi^2^ = 4.77; *p* = 0.31).

#### Wound irritation

**Figure 4.**
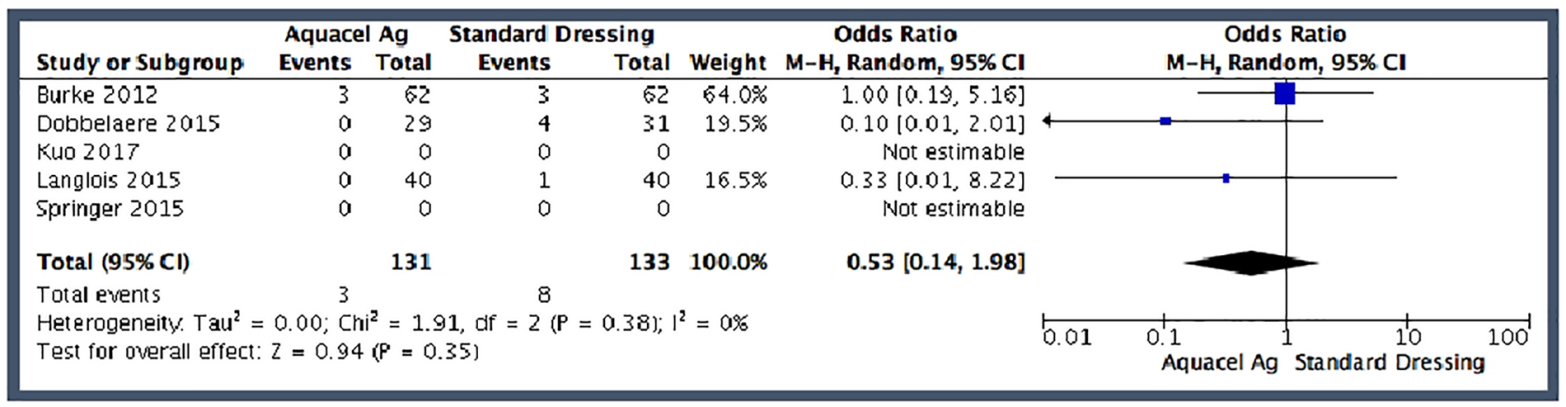
Pooled analysis for wound irritation.

3 of the 5 studies evaluated wound irritation. None of the studies defined exactly what was classified as wound irritation. The pooled risk estimates of wound irritation were 2.2% in the hydrofibre dressing group (*n* = 3 of 131) and 6.0% in the standard dressing group (*n* = 8 of 133). The pooled effect estimate demonstrated no significant difference between the treatment groups for wound irritation (OR 0.53; 95% CI, 0.14–1.98) (Figure 4). There was no heterogeneity across the studies (I^2^ = 0%; chi^2^ = 1.91; *p* = 0.38).

#### Dressing changes

**Figure 5.**
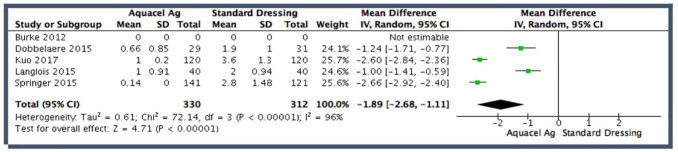
Pooled analysis for number of dressing changes.

4 of the 5 studies evaluated the number of dressing changes. The pooled effect estimate showed a statistically significant reduction in dressing changes associated with the hydrofibre dressing (MD 1.89, 95% CI, 1.11–2.68) (Figure 5). However, there was considerable heterogeneity across the studies (I^2^ = 96%) which was statistically significant (chi^2^ = 72.1, *p* < 0.00001).

## Discussion

From a clinical effectiveness standpoint, the evidence for hydrofibre dressing remains equivocal when it comes to the most important outcome of PJI due to low event rates in all groups. Furthermore, the impact on wound irritation is unclear. Hydrofibre dressings do seem to improve short-term outcomes including wound blistering and reduce the number of dressing changes required. However, in some of the trials, the criteria for dressing changes were not equivalent across groups. This inherently biased treatment effect to favour the hydrofibre dressings. Furthermore, the conclusions are also limited by the general poor methodological quality and high risk of bias inherent in the studies.

Interestingly, only 1 deep infection event was reported across all studies in either group. This is likely due to the limited follow-up period for most of the trials. Thus, it is difficult to assess the efficacy of hydrofibre dressing on rates of PJI, which is the most important infectious complication of TJA. In general, few high quality studies exist that assess the efficacy of the plethora of available wound dressings.^17^ A recent systematic review and meta-analysis of randomised controlled trials analysed the impact of incisional negative-pressure wound therapy (NPWT) in total joint arthroplasty.^18^ They found that while NPWT had no impact on total infection or deep infection rates, it did reduce superficial infection rates (OR 0.19; 95% CI, 0.04–0.90; *p* = 0.04). Given similar findings in our study, this may suggest that simply keeping the wound covered with an advanced type dressing is sufficient at achieving a reduction in superficial infection. It is unclear if the actual negative pressure component has any added benefit.

The strengths of this review stem from its thorough and stringent methodology. However, there are limitations to the current review. First and foremost, although all the included trials evaluated the same type of hydrofibre dressing as the intervention, they varied in the type of dressing used in the control arm (“standard dressing”). Despite the variation, all trials used standard dressings in their control arm which generally consisted of some form of gauze pad and an adhesive. In addition, some trials used a silver-impregnated dressing as their intervention, while others used the same dressing but with no silver. Secondly, despite the inclusion of exclusively, recently published RCTs to ensure the best currently available evidence on the topic, the trials included were generally of poor methodological quality with limited follow-up periods. Finally, we did not evaluate patient-reported outcomes such as patient satisfaction and pain.

## Conclusion

In conclusion, evidence suggests hydrofibre dressings have no observable effect on PJI and wound irritation. Evidence for reduction in blisters and number of dressings is modest given wide confidence intervals and biased trial methodologies. Use of hydrofibre dressings should be considered inconclusive for mitigating major complications in light of current best evidence.

## Appendix

**Table 1.** Study characteristics.

Study	Country	Age (years)	Sample size	Male %	Procedure	Intervention	Control
Burke et al.^12^	Ireland	67	124	62	THA and TKA	Aquacel	Mepore
Dobbelaere et al.^13^	Belgium	63	111	37	TKA	Aquacel surgical	Cosmopor E
Kuo et al.^14^	Taiwan	70	240	27	TKA	Aquacel Ag surgical	Sofra-Tulle + Gauze + Tape
Langlois et al.^15^	France	70	80	30	THA and TKA	Aquacel	Gauze + Crepe Bandage
Springer et al.^16^	USA	72	262	45	THA and TKA	Aquacel Ag surgical	Primapore

**Table 2.** Study outcomes.

Study	Follow-up	Treatment	Blisters (%)	Wound irritation (%)	Infection	Mean dressing changes (Mean, SD)
Burke et al.^12^	To hospital discharge	Aquacel	3/62 (4.8)	3/62 (4.8)	0/62	N/A
		Mepore	11/62 (17.7)	3/62 (4.8)	0/62	N/A
Dobbelaere et al.^13^	5 days	Aquacel	2/29 (6.9)	0/29 (0)	0/29	0.66 (0.85*)
		Cosmopor E	0/31 (0)	4/31 (12.9)	0/62	1.9 (1*)
Kuo et al.^14^	1 year	Aquacel	3/120 (2.5)	N/A	0/120	1 (0.2)
		Sofra-Tulle + Gauze + Tape	6/120 (5)	N/A	1/120	3.6 (1.3)
Langlois et al.^15^	To hospital discharge	Aquacel	0/40 (0)	0/40 (0)	0/40	1 (0.91)
		Gauze + crepe bandage	1/40 (2.5)	1/40 (2.5)	0/40	2 (0.94)
Springer et al.^16^	1 month	Aquacel	1/141 (0.7)	N/A	0/141	0.14 (0)
		Primapore	7/121 (5.8)	N/A	0/121	(1.48)

* SD, standard deviation-obtained from Sharma et al.^19^
